# A nonlinear creep model of hard structural planes

**DOI:** 10.1371/journal.pone.0315586

**Published:** 2024-12-12

**Authors:** Aneng Cui, Yongxin Dai, Chao Jia, Quansheng Mao, Kelin Yu, Pengcheng Wu, Mengsheng Zhao

**Affiliations:** 1 Sinosteel Maanshan General Institute of Mining Research CO.,Ltd., Maanshan, China; 2 Institute of Marine Science and Technology, Shandong University, Qingdao, China; 3 Huawei National Engineering Research Center for Efficient Recycling of Metallic Mineral Resources Co.,Ltd., Maanshan, China; 4 Key Laboratory of Disaster Prevention and Control for Non-coal Open-pit Mines, Maanshan, China; University of Perugia: Universita degli Studi di Perugia, ITALY

## Abstract

Hard structural planes mainly exist in rock slopes and their creep characteristics largely determine slope stability. Traditional models have some shortcomings in describing the creep characteristics of hard structural planes, such as poor adaptability and unclear physical meaning of parameters. In order to overcome these shortcomings, based on the creep failure mechanism of hard structural planes, an element combination model is adopted in the study. In the instantaneous deformation stage, the plastic deformation proportional coefficient *n* is introduced based on the strain rebound theory of loading-unloading tests. In the attenuation creep stage, the hardening coefficient *C* and creep index *m* are introduced. In the viscoelastic-plastic failure stage, the weakening factor *k* is introduced. By improving traditional elements, a new piecewise nonlinear constitutive relationship of hard structural planes is established and then the creep equation is obtained with integration method. The adaptability of the established model and the way to solve parameters are analyzed and the correctness of the model is proved theoretically. The data of creep tests of the prefabricated serrated interpenetrated green sandstone structural plane and the concealed non-interpenetrated marble structural plane are further fitted and verified, yielding a fitting result exceeding 0.95, thereby indicating a strong correlation. By optimizing the whole creep process of the hard structural plane in stages and demonstrating the difference in the creep mechanism of the hard structural plane at different depths in a rock mass in the high and low stress fields in the form of piecewise function, the physical meaning of the improved model is clearer. In addition, the improved model allows the higher accuracy of nonlinear characteristics in attenuation creep stage and acceleration creep stage and provides the theoretical basis for the stability analysis of rock slopes.

## Introduction

The creep property of a rock mass is one of the main factors affecting the time-dependent stability of rock slopes [1]. Compared with the obvious creep characteristics of a soft rock slope, the insignificant creep characteristics of the hard structural plane in a rock slope largely determine the deformation and long-term strength of the rock mass [2]. The overall stability of a rock slope is mainly related to the tendency of structural planes and a creep failure most easily occurs in a gently inclined layered rock slope because the creep deformation of the upper and lower plates of the large-scale through rock structure may lead to interlayer dislocation [3]. At present, the creep characteristics of hard structural planes at home and abroad have been explored in the following two aspects [4–6]. Firstly, due to the influence of mining disturbance on the structural plane exposed in engineering practices, it was difficult to ensure the integrity of the upper and lower plates of the structural plane. Most of existing results were obtained based on a prefabricated regular serrated interpenetrated structural plane. The influences of basic friction angle, roughness and climbing angle on the creep characteristics of a hard structural plane were investigated. Secondly, with a rock mass with the concealed non-interpenetrated structural plane as the object, the dynamic evolution of the strain in the whole process from the first development of hard structural plane to the final interpenetrated failure was studied.

In 1936, Griggs [7] proposed a rock creep constitutive model related to limestone and shale. In general, current research objects are mainly intact rock or soft soil. Compared with intact rock or soft rock, the hard structural plane showed the complicated creep characteristics due to the influences of penetration type, surface morphology, roughness, weathering degree, undulation, and moisture content [8]. Shen Mingrong et al. [9] prepared regular serrated interpenetrated structural planes with cement mortar and studied the time-dependent variations of climbing angle, stress, and strain of structural planes with different climbing angles under different normal stress conditions. Liu [10] explored the influences of the tooth height of structural plane on strain, rate, failure mode, and failure time under different stress levels and analyzed the failure mechanism of serrated joint rock. Based on the study on long-term strength characteristics of regular serrated structural planes, Zhang et al. [11] proposed an empirical formula for shear creep of rock structural planes.

A prefabricated serrated interpenetrated structural plane used as the sample could reduce the complexity of a structural plane and the discreteness of results. However, the regular serrated structural plane does not have acceleration creep characteristics [12] and is inconsistent with the progressive loss-of-stability failure mechanism of the rock slope controlled by a structural plane [13]. The creep characteristics of non-interpenetrated structural planes have been explored by some scholars. Li et al. [14] established a creep damage constitutive model based on the stage characteristics of creep curves of concealed non-interpenetrated structural planes. Zhang Fengrui [15] carried out the shear creep test of granite natural structural plane in the multi-environment and obtained the creep model of rock mass structural plane under different normal stress conditions. However, due to the obvious heterogeneity, anisotropy, and discontinuity of hard structural planes [16] as well as the difficulty in acquiring samples with similar characteristics in engineering practices, the traditional creep model is not suitable to describe the creep characteristics of hard structural planes [17].

The study aims to explore the phenomenon of shear creep failure of rock mass along the structural plane. The hard structural plane is the main controlling factor of the long-term stability of a rock slope. In the study, based on the creep mechanism of the hard structural plane in each creep stage, through improving traditional elements, a nonlinear creep model of hard structural plane is proposed by means of element combination. The proposed model can describe the variations of stress and strain in the three creep stages and the nonlinear characteristics of the hard structural plane in attenuation creep stage and acceleration creep stage.

## Creep failure mechanism of hard structural planes

### Creep failure of a serrated interpenetrated structural plane

**Fig 1:**
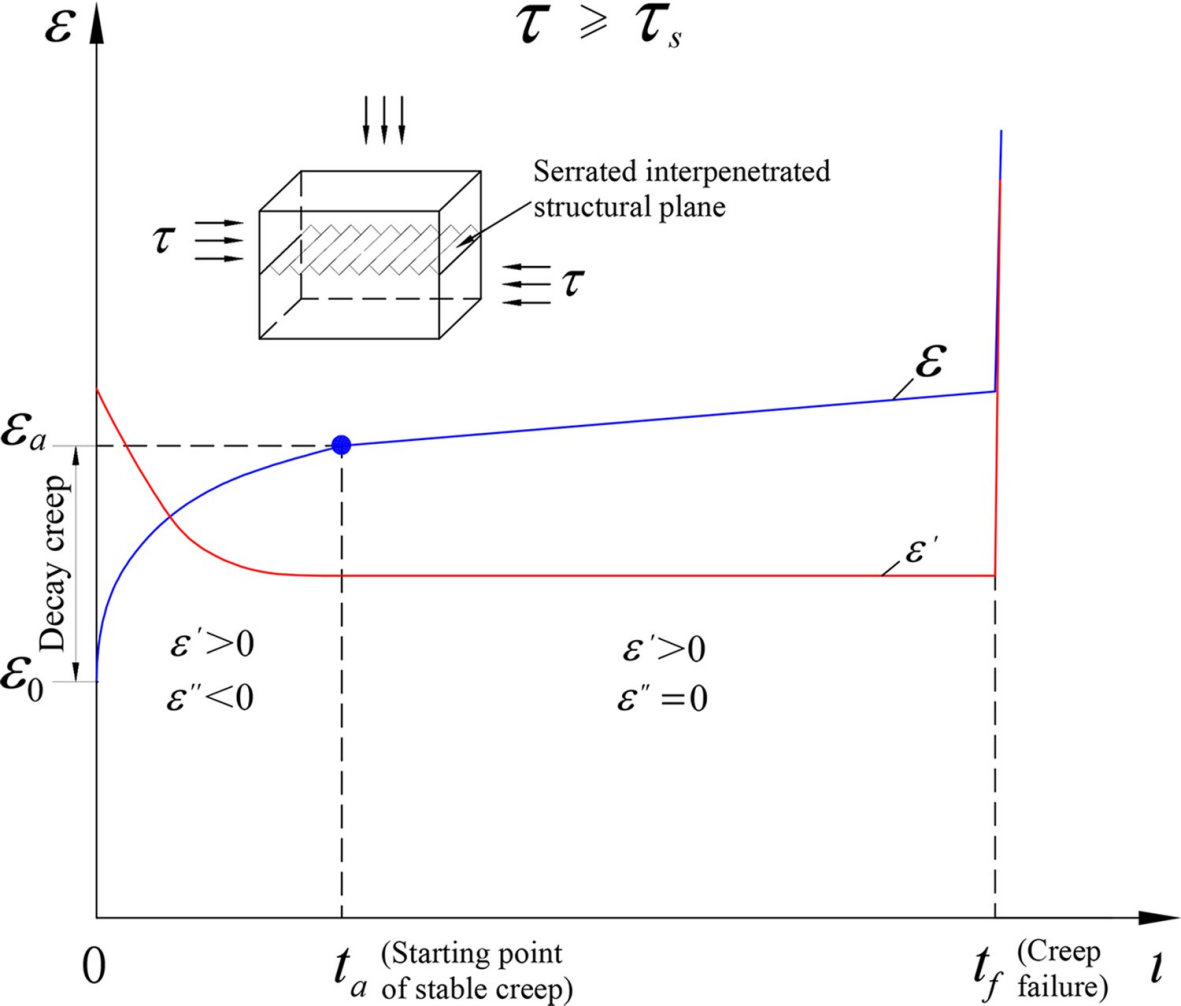
Creep curve of a serrated interpenetrated structural plane.

The surface morphology of a rock structural plane is one of the main factors affecting its creep characteristics [18]. The serrated interpenetrated structural plane is generally obtained by pouring with cement mortar or cutting a complete rock block into the upper and lower plates and mainly used to explore the time-dependent variations of climbing angle, stress, and strain under different normal stress conditions. A filler does not exist in the hard structural plane, so the interbedding phenomenon of soft and hard rock mass does not occur. Under the action of compression and shear stress, the strength of a hard structural plane is not much different from that of upper and lower blocks. The creep characteristics of the prefabricated serrated interpenetrated structural plane are similar to those of hard rock and not obvious under the condition of low stress level, but long-term mechanical properties can be observed under a high stress [19]. The structural plane in the shallow part of the rock mass is in a low stress environment and only an attenuation creep may occur. The structural plane in the deep part of the rock mass is in a high stress environment and an isometric creep may occur. When the structural plane of rock mass is destroyed, the structural plane belongs to the viscoplastic failure mode. As shown in Fig 1, due to the large elastic modulus of cement mortar and intact rock, the instantaneous elastic deformation (*ε*_0_) under loading is small. After the elastic stage, the creep rate (*ε’*) of the hard structural plane gradually decreases, showing creep hardening characteristics of attenuation creep stage and constant creep stage. When the stress level exceeds its long-term strength (*τ*_s_), the sliding failure of the structural plane has obvious instantaneity in the later constant creep stage and does not show the nonlinear characteristics of the acceleration creep stage. The above results are interpreted as follows. The creep deformation of the interpenetrated structural plane is mainly affected by climbing angle and friction force of the serrated structure and there is no obvious process of crack development and expansion.

### Creep failure of a concealed non-interpenetrated rock structural plane

**Fig 2:**
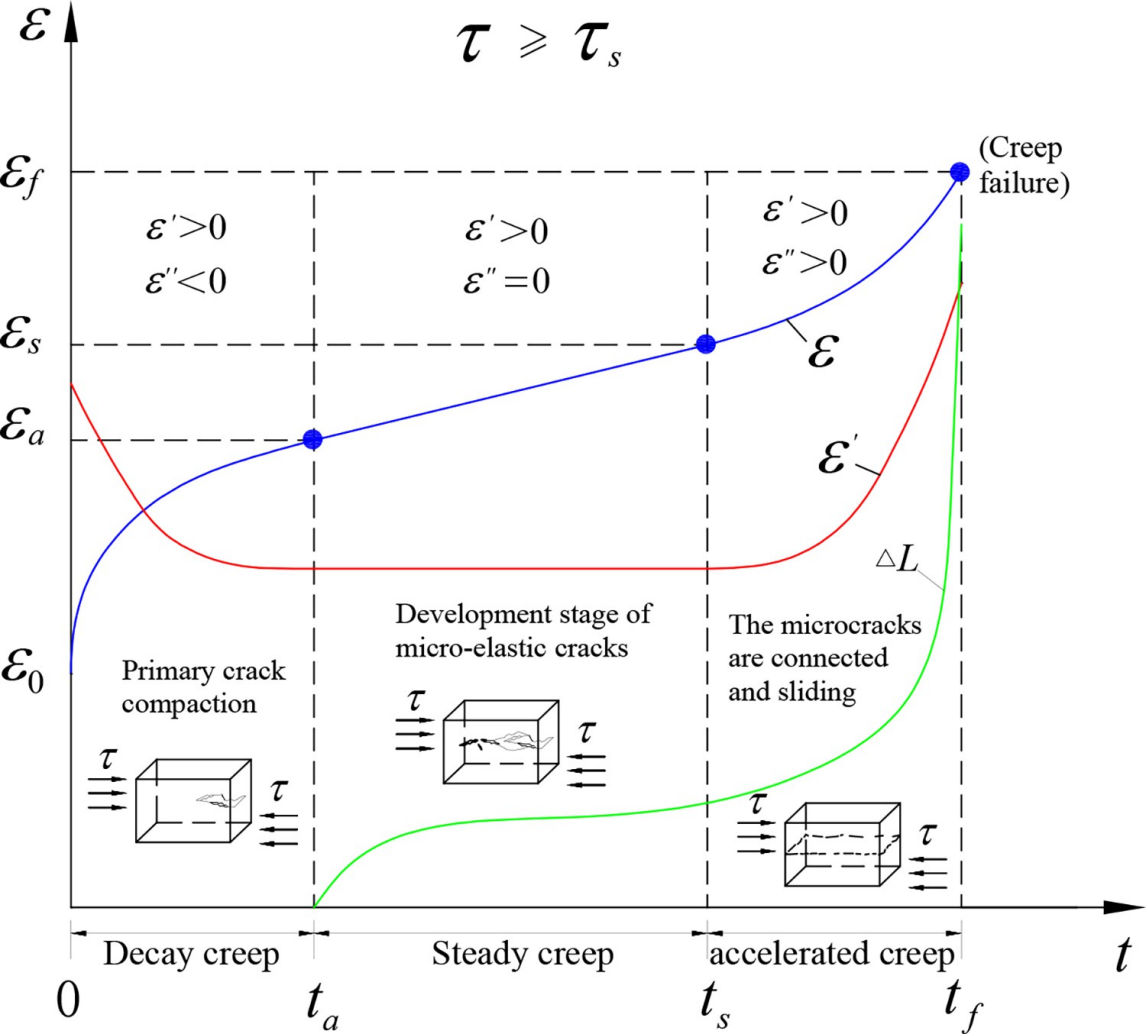
Creep curve of a concealed non-interpenetrated structural plane.

A concealed non-interpenetrated structural plane refers to a structural plane that exists inside a rock mass and is surrounded by a complete rock bridge [20]. Based on the damage failure mechanism [21], under the action of a stress field, the non-interpenetrated structural plane presents the creep failure characteristics of crack closure-crack development-gradual penetration of structural plane [22]. When the stress level exceeds its long-term strength, such a structural plane exhibits the complete three-stage creep characteristics (Fig 2). The stress level required for creep deformation of rock mass has upper and lower thresholds. For example, when the stress environment of rock mass reaches 40% ~ 80% of its long-term strength, the strain gradually changes. When the stress level is higher than the lower limit of the stress threshold required for creep, in the initial loading stage, cracks in the rock mass are gradually closed and produce irreversible instantaneous plastic deformation. The elastic-plastic deformation rate decreases gradually before the crack expands. The length of the main structural plane L remains unchanged and there is no new damage, showing the phenomenon of creep hardening. Under the continuous action of a high stress, the length L of the structural plane extends gradually along the direction of the main crack and the strain increases steadily, showing the obvious viscoelastic plasticity. When the stress level is higher than the upper limit of the stress threshold required for creep, the faster the propagation rate of the main crack of the structural plane is, the stronger the plasticity of the structural plane deformation and the weaker the viscosity is. In the process from the continuous expansion of the structural plane to the macroscopic penetration, the creep rate and the plastic strain ratio increases and the viscous strain ratio decreases sharply. The acceleration creep stage of the non-penetrating structural plane has the nonlinear characteristics, but the stage is short, showing the weakening phenomenon of material damage.

### Piecewise-nonlinear creep model

According to the creep failure mechanism of hard structural plane in each stage, traditional creep elements were improved and a highly adaptive creep model of structural plane was established in the form of element combination [23].

### Instantaneous strain

Compared with rocks, the hard structural plane contains the more obvious gap between upper and lower plates. In the instantaneous elastic-plastic deformation stage during stress loading, the hard structural plane produces the more instantaneous plastic strain [24]. The instantaneous plastic strain can be obtained with the difference between the stress-strain curves of instantaneous loading-unloading tests. When the stress level of sandy shale is lower than its long-term strength [25], the ratio of instantaneous elastic strain to plastic strain is about 7:1 to 4:1. The instantaneous strain approximately increases linearly with the increase in stress level, whereas the strain difference decreases with the increase in the load, indicating the limited plastic deformation. In order to characterize the failure mechanism of the irreversible instantaneous plastic deformation caused by the gradual closure of the cracks in the interlayer of the structural plane, the initial elastic-plastic proportional coefficient *n* is introduced and the instantaneous strain of the hard structural plane is simplified into a linear function related to the stress level so as to improve the traditional elastic element **H**. The improved instantaneous element ***n*-H** is expressed as:

$$\begin{array}{l} \varepsilon_{0}=\varepsilon_{e}+\varepsilon_{p}\\ \varepsilon_{e}=n\varepsilon_{p} \end{array}\}$$ (1)

where *ε*_0_ is the instantaneous total strain; *ε*_*e*_ is the instantaneous elastic strain; *ε*_*p*_ is the instantaneous plastic strain.

Based on Eq (1), we get:

$$\frac{\tau }{G_{e}}=n\frac{\tau }{G_{p}}$$ (2)

where *G*_*e*_ is the elastic modulus; *G*_*p*_ is the plastic modulus.

Thus, instantaneous total strain is improved as:

$$\varepsilon =(1+\frac{1}{n})\frac{\tau }{G_{1}}$$ (3)

where *G*_1_ is the elastic modulus of instantaneous element ***n*-H**.

### Attenuation creep stage

The viscosity of the Newtonian body in the traditional viscous element **N** is fixed [26], so it fails to reflect the creep mechanism of the hard structural plane in the attenuation creep stage when fine cracks are gradually closed and the strain rate gradually decreases and becomes stable. At this stage, the strain rate of the hard structural plane is negatively correlated with the strain, showing a certain hardening characteristics, and also positively correlated with the stress level:

$$\dot{\varepsilon }=f(\tau ,\varepsilon )=\frac{F(\tau )}{Q(\varepsilon )}$$ (4)

Based on the hardening characteristics of the hard structural plane in the attenuation creep stage, the element **N** is improved. It is assumed that the viscosity coefficient of the hard structural plane is a composite function of stress and time:

$$\eta (\tau ,t)=\eta_{1}\langle C\rangle {\left(\frac{\tau }{\tau_{v}}\right)}^{m}{\left(\frac{t}{t_{v}}\right)}^{1-m}$$ (5)

where *τ*_*v*_ and *t*_*v*_ are the standard reference stress and reference time and equal 1; *m* is the creep index; *η*_1_ is the viscosity coefficient of attenuation creep stage; <*C>* is the Heaviside step function. When *t* = 0, the initial value of the viscosity coefficient is a constant related to the stress level. When *t* > 0, the viscosity decreases and the plasticity increases. *C* is the hardening coefficient.


$$\langle C\rangle =\{\begin{array}{l} 1,t=0\\ C,t>0 \end{array}$$ (6)

The improved viscous element *C*-**N** is used to replace the traditional element in the Kelvin body as follows:

$$\tau =G_{2}\varepsilon +\eta_{1}(\tau ,t)\frac{d\varepsilon }{dt}$$ (7)

Based on Eqs (5) to (7), after solving the differential equation by separating variables, the improved creep equation of the Kelvin body is obtained as follows:

$$\varepsilon =\frac{\tau }{G_{2}}+A\exp(-\frac{G_{2}t^{m}}{Cm\eta_{1}\tau^{m}})$$ (8)

When *t* = 0, the instantaneous strain does not occur in the viscous body, namely, *ε* = 0. Then, we get:

$$\varepsilon =\frac{\tau_{0}}{G_{2}}-\frac{\tau_{0}}{G_{2}}\exp(-\frac{G_{2}t^{m}}{Cm\eta_{1}\tau^{m}})$$ (9)

### Constant creep stage and acceleration creep stage

When the stress level *τ*_0_ of the hard structural plane is less than its long-term strength *τ*_s_, the creep rate gradually approaches zero and can be characterized by a dashpot element with a constant viscosity. When *τ*_0_ ≥ *τ*_s_, the increasing trend of strain turns from the linear growth state into the nonlinear acceleration growth state at t^*^ until the occurrence of viscoplastic failure. The higher stress level corresponds to the faster viscoplastic failure and the larger creep acceleration of the hard structural plane, showing the accelerated weakening characteristics of viscosity with the increase in stress level and time. Thus, it is assumed that the viscosity coefficient of the hard structural plane at this stage is a composite exponential function of stress and time. In this way, a new nonlinear viscous element *k*-**N** is established as:

$$\eta \left(\tau ,t\right)=\eta_{2}[1+\frac{1}{t}\exp(-k\tau t)]$$ (10)

where *k* is the weakening coefficient related to the material properties of the hard structural plane.

By the combination of the element *k*-**N** and the plastic element, a new nonlinear viscoplastic element is obtained as:

$$\tau =\{\begin{array}{l} \dot{\varepsilon }\eta_{2},t<t^{*}\\ \dot{\varepsilon }\eta_{2}\left[1+\frac{1}{t}\exp(-k\tau t)\right],t\geq t^{*} \end{array}$$ (11)

When *τ*_0_ ≥ *τ*_s_, the improved creep equation of nonlinear viscoplastic element is obtained by solving the above differential equation:

$$\varepsilon =\{\begin{array}{l} \frac{\left(\tau -\tau_{s}\right)}{\eta_{2}}t,t<t^{*}\\ \frac{\left(\tau -\tau_{s}\right)}{\eta_{2}}t+\frac{(\tau -\tau_{s})kt-1}{\eta_{2}(\tau -\tau_{s})k^{2}}\exp[(\tau -\tau_{s})kt],t\geq t^{*} \end{array}$$ (12)

### Improved nonlinear viscoelastic-plastic creep model (*n*-*C*-*k*-NVEP Model)

**Fig 3:**
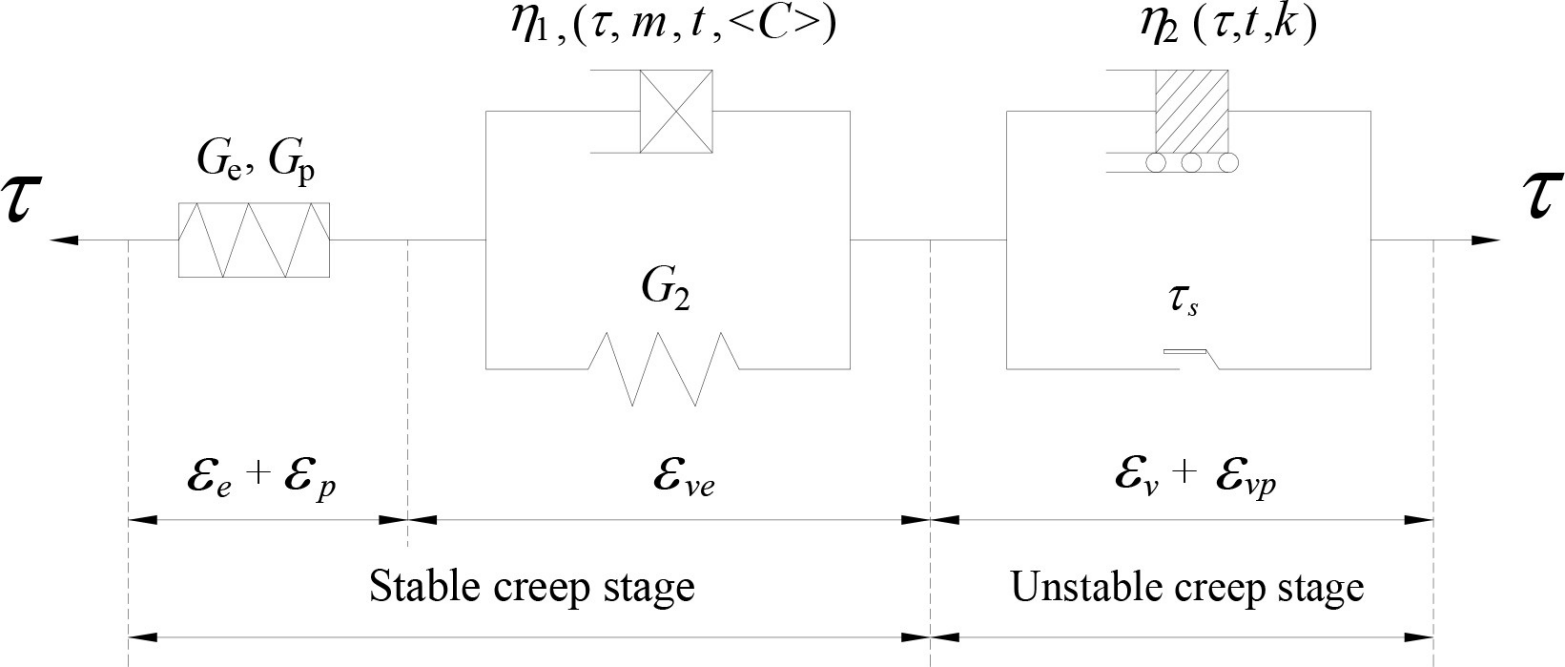
*n*-*C*-*k*-NVEP model.

According to two creep stages (stable and unstable stages), the *n*-*C-k*-NVEP model is obtained by combining the two creep stages in a segmented and series manner. The model diagram is shown in Fig 3.

Firstly, the improved constitutive relation of traditional elements is established and then the creep equation of this part is obtained with integration method. Then, the overall creep equation of *n-C-k*-NVEP Model is obtained with superposition method [27]:

$$\varepsilon =\{\begin{array}{l} (1+\frac{1}{n})\frac{\tau_{0}}{G_{1}}+\frac{\tau_{0}}{G_{2}}-\frac{\tau_{0}}{G_{2}}\exp(-\frac{G_{2}t^{m}}{Cm\eta_{1}\tau^{m}}),\tau_{0}<\tau_{s}\\ (1+\frac{1}{n})\frac{\tau_{0}}{G_{1}}+\frac{\tau_{0}}{G_{2}}-\frac{\tau_{0}}{G_{2}}\exp(-\frac{G_{2}t^{m}}{Cm\eta_{1}{\tau_{0}}^{m}})+\frac{\left(\tau_{0}-\tau_{s}\right)}{\eta_{2}}t,\tau_{0}\geq \tau_{s},t\leq t^{*}\\ (1+\frac{1}{n})\frac{\tau_{0}}{G_{1}}+\frac{\tau_{0}}{G_{2}}-\frac{\tau_{0}}{G_{2}}\exp(-\frac{G_{2}t^{m}}{Cm\eta_{1}{\tau_{0}}^{m}})+\frac{\left(\tau_{0}-\tau_{s}\right)}{\eta_{2}}t+\frac{{\left(\tau_{0}-\tau_{s}\right)}^{2}k^{2}-1}{\eta_{2}(\tau_{0}-\tau_{s})k^{2}}\exp[(\tau_{0}-\tau_{s})kt],\tau_{0}\geq \tau_{s},t>t^{*} \end{array}$$ (13)

## Adaptive analysis

### Instantaneous strain stage

In the improved element expressed in Eq (3), the instantaneous elastic-plastic relationship of the material of the hard structural plane is related to the coefficient *n*. The larger the *n* is, the larger the initial instantaneous plastic strain is. The proportional coefficient *n* is related to the elastoplastic difference between the hard structural plane and the soft rock with obvious creep characteristics at the initial stress loading stage. In this way, the physical meaning of the model is clearer.

### Attenuation creep stage

According to Eq (9), the strain rate of the attenuation creep stage is:

$$\varepsilon^{'}=\frac{\tau_{0}^{1-m}t^{m-1}}{C\eta_{1}}\exp(-\frac{G_{2}t^{m}}{Cm\eta_{1}\tau_{0}^{m}})$$ (14)

When *C* > 0, *ε*^’^ > 0, indicating the strain rate is greater than zero.

According to Eq (14), the creep acceleration at the attenuation stage is:

$$\varepsilon^{"}=\frac{\tau_{0}^{1-m}t^{m-2}}{C\eta_{1}}[(m-1)-\frac{\tau_{0}^{-m}t^{m}G_{2}}{C\eta_{1}}]\exp(-\frac{G_{2}t^{m}}{Cm\eta_{1}\tau_{0}^{m}})$$ (15)

**Fig 4:**
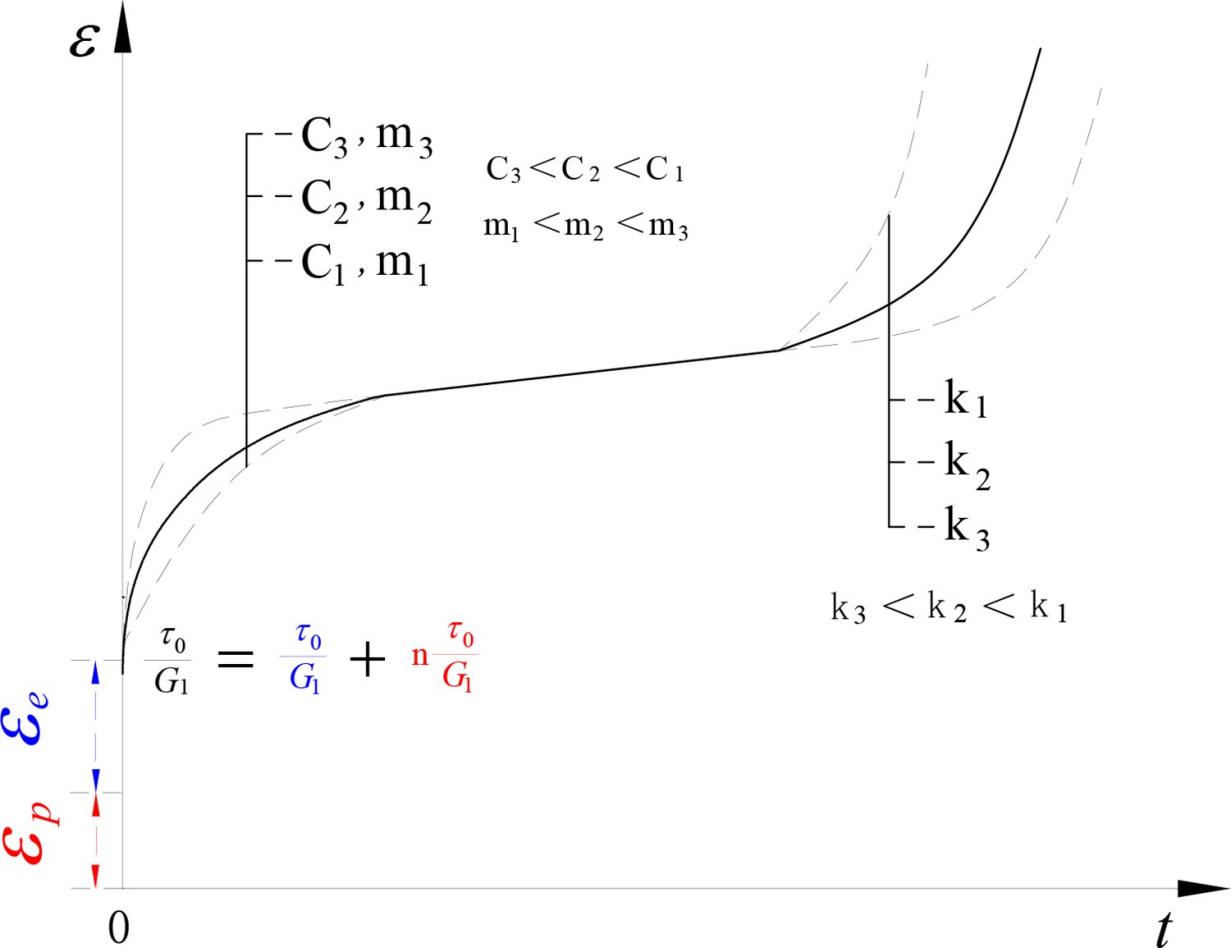
Comparison of different creep curves.

When the creep index *m* ≤ 1, *ε*^’^ < 0. In other words, the creep rate in the attenuation creep stage gradually decreases. Eq (15) can describe the characteristics of increasing strain and decreasing creep rate in the attenuation creep stage. The larger the hardening coefficient *C* is, the faster the decrease of creep rate is. The larger the creep index *m* is, the slower the decrease of creep rate is, as shown in Fig 4.

### Constant creep stage and acceleration creep stage

According to Eq (12), we get:

$$\varepsilon^{'}=\frac{\tau_{0}-\tau_{s}}{\eta_{2}}t\exp[kt(\tau_{0}-\tau_{s})]$$ (16)

The stress field of the hard structural plane is different at different depths in the rock slope. When *τ*_0_ = *τ*_s_, *ε*^’^ = 0 is obtained from Eq (16), indicating that the creep rate is constant.

When *τ*_0_ > *τ*_s_, Eq (16) is greater than zero, indicating that the strain rate is larger than zero.

According to Eq (16), we get:

$$\varepsilon^{"}=\frac{\tau_{0}-\tau_{s}}{\eta_{2}}[1+tk(\tau_{0}-\tau_{s})]\exp[k(\tau_{0}-\tau_{s})t]$$ (17)

When *k* ≥ 0, *ε*^’^ > 0. Eq (17) can describe the characteristics of increasing deformation and increasing creep rate in the acceleration creep stage. The larger *k* corresponds to the larger creep acceleration and the shorter duration of the acceleration creep stage.

## Identification of model parameters

The most commonly used identification method of model parameters is the least squares fitting method [28]. The *n-C-k*-NVEP Model has 8 parameters to be identified: *n*, *G*_1_, *m*, *C*, *η*_1_, *G*_2_, *η*_2_, and *k*. The least squares fitting method is greatly affected by the initial reference values of the parameters to be identified. It is difficult to carry out the fitting analysis directly and direct fitting is prone to yield the poor correlation. The above analysis results are summarized below. Firstly, the initial values (*n*^’^ and *G*_1_^’^) of the plastic strain scale factor ***n*** and the elastic modulus *G*_1_ can be determined in the loading-unloading test of the hard structural plane. Secondly, *η*_1_ affects the creep rate at the attenuation creep stage and the strain at this stage finally becomes stable. A large initial value *η*_1_^’^ can be selected and the simplified strain trend of the attenuation creep stage has little effect on the fitting results of the complete creep curve [29]. Under the fixed condition of *n*^’^, *G*_1_^’^, and *η*_1_^’^, with the optimal inversion method, the objective function is designed to identify the remaining five parameters with the optimal value of the residual sum of squares of actual displacement and calculated displacement. The optimized model parameters are expressed as:

$$x=(m,C,G_{2},\eta_{2},k)=(x_{1},x_{2},\ldots ,x_{5})$$ (18)

Then, with the nonlinear function *y* = *f* (*x*, *d*), the solution process is transformed into the iterative calculation of the least squares method. The residual sum of squares *e* is calculated with P (*x*_i_, *y*_i_):

$$e=\sum_{i=1}^{p}{\left[Y_{i}-f(x_{i},d)\right]}^{2}$$ (19)

where *x* is the independent variable vector; *d* is the unknown parameter vector; *f* (*x*_i_, *y*_i_) is the calculated displacement value at time *i*. In order to ensure the rationality of the identification results of model parameters, the upper and lower limits of the parameters to be fitted can be set according to the test results of similar structural planes. The inversion convergence criterion is expressed as:

$${\left\{\frac{1}{l}\sum_{i=1}^{l}{\left[f(X_{1})-f(X_{v})\right]}^{2}\right\}}^{\frac{1}{2}}\leq \varepsilon$$ (20)

where *X*_1_ is the optimal solution under the design requirements and *X*_v_ is the target point of function.

## Experimental verification

**Fig 5:**
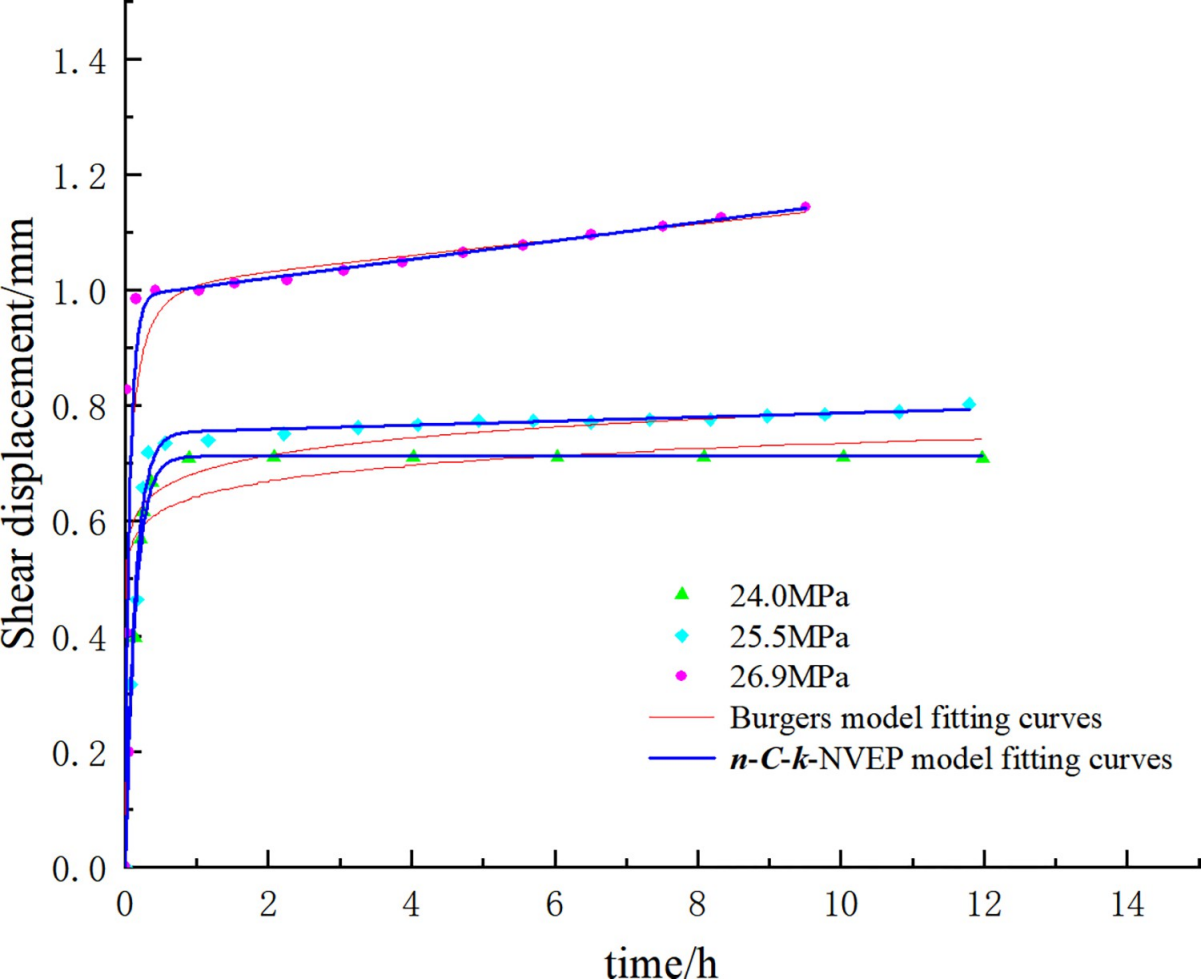
Creep test data and fitting results of serrated interpenetrated green sandstone structural plane.

**Fig 6:**
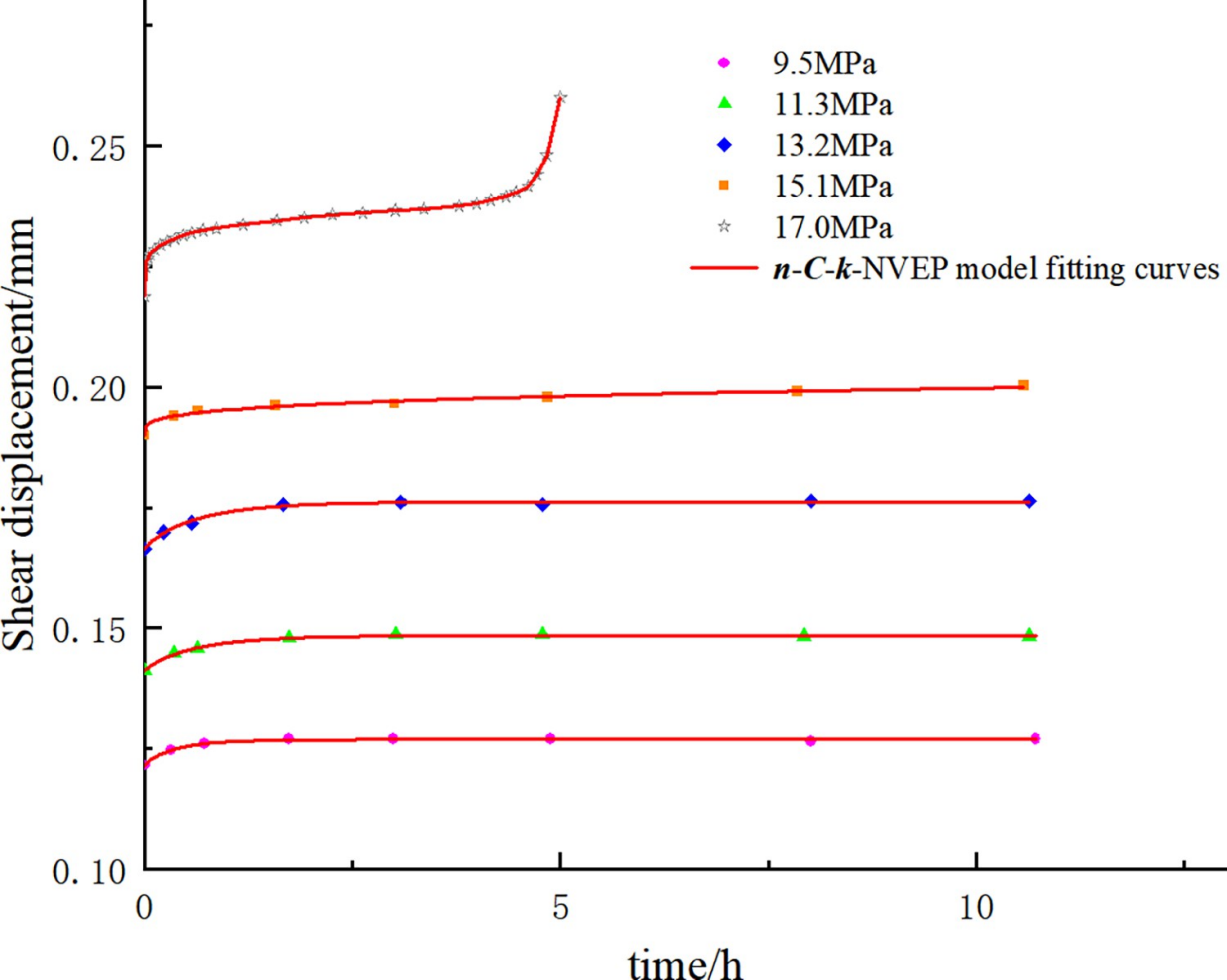
Creep test data and fitting results of concealed non-interpenetrated marble structural plane.

The applicability of the *n*-*C*-*k*-NVEP model is further verified with the experimental data of the serrated interpenetrated structural plane and the concealed non-interpenetrated structural plane. In the previous study [30], the parameters of the green sandstone structural plane were set as follows: the sample size (50 mm * 50 mm * 58 mm), the serrated angle (45°), tooth height (5 mm). The creep test data under the three shear stress levels (0.85 *τ*_max_, 0.90 *τ*_max_, and 0.95 *τ*_max_, *τ*_max_ = 28.23 MPa, the peak stress of direct shear test) are shown in Fig 5. In another previous study [31], the sample size of the concealed non-interpenetrated marble structural plane was set as 100 mm * 100 mm * 100 mm. The shear creep test data under the staged continuous loading condition and the normal stress of 15 MPa are shown in Fig 6.

The fitting results are shown in Figs 5 and 6 and Tables 1 and 2. Firstly, the long-term strength of the serrated interpenetrated green sandstone structural plane is close to 90% ***τ***_max_. Compared with the Burgers model, the *n-C-k*-NVEP model adjusts the viscosity of the element through the hardening coefficient *C* in order to adapt to the change of creep rate under different stress levels and yields the higher fitting accuracy in the attenuation creep stage. In this way, the poor adaptability of the traditional creep element in the attenuation creep stage is improved. Secondly, the fitting degree of creep test data of concealed non-interpenetrated marble structural plane is high, indicating that the model can well describe the complete three-stage creep characteristics of the concealed non-interpenetrated rock structural plane and solve the deficiency of the traditional creep model in describing the nonlinear characteristics of acceleration creep stage.

**Table 1:** Fitting results of the creep model of serrated interpenetrated green sandstone structural plane.

Stress/MPa	*G*_1_/MPa	*G*_2_/MPa	*η*_1_/MPa·h	*η*_2_/MPa·h	*n*	*m*	*C*	*R* ^2^
24.0	35.129	499.613	1507.661	7161.386	3.374	0.751	14.552	0.992
25.5	33.915	433.175	1478.023	7319.122	3.381	0.732	15.379	0.991
26.9	32.061	480.127	1341.089	7667.175	3.388	0.673	17.618	0.961

**Table 2:** Fitting results of the creep model of the concealed non-interpenetrated marble structural plane.

Stress/MPa	*G*_1_/GPa	*G*_2_/GPa	*η*_1_/GPa·d	*η*_2_/GPa·d	*n*	*m*	C	*k*	*R* ^2^
9.5	72.976	1767.433	863.221	1186.273	3.461	0.913	10.126	-	0.995
11.3	70.410	1296.581	835.363	1113.767	3.488	0.896	11.491	-	0.991
13.2	61.011	1352.495	820.251	1179.331	3.476	0.886	11.633	-	0.992
15.1	79.798	1906.736	872.879	1157.416	3.493	0.811	11.616	-	0.979
17.0	69.730	1872.653	859.001	1206.911	3.392	0.777	12.895	0.225	0.993

## Conclusion

Based on the deformation mechanism of the hard structural plane in different creep stages, the traditional elements are improved and the *n-C-k*-NVEP model is obtained in the form of element combination. The obtained model has higher fitting accuracy at the attenuation creep stage and can describe the nonlinear characteristics of the acceleration creep stage.

The whole creep process of the hard structural plane is optimized in stages. The piecewise function is used to describe the difference in the creep mechanism of the hard structural plane at different depths in the rock mass under the action of high or low stress fields. The physical meaning of the improved model parameters is clearer.

Through the stability analysis of the model and the fitting calculation with the creep test results of the serrated interpenetrated and concealed non-interpenetrated rock structural planes, the applicability of the model is verified from both theoretical and experimental aspects.

The creep characteristics of a hard structural plane are the internal cause for rock slope instability. Its constitutive relationship contributes to numerical simulation and provides the theoretical basis for the instability prediction and early warning of rock slope.
